# Academy of Stomatology

**Published:** 1898-06

**Authors:** 

**Affiliations:** Academy of Stomotology


### ACADEMY OF STOMATOLOGY.

A regular meeting of the Academy of Stomatology, of Philadelphia, was held January 25, 1898, Dr. Edwin T. Darby in the chair.

A paper on "The Preparation of the Mouth for an Artificial Denture preparatory to taking an Impression" was read by Professor Robert H. Nones.

(For Professor Nones's paper, see page 270.)

### DISCUSSION.

*Dr. H. B. Hickman.*—I should like to know what Dr. Nones would use for cleaning the rubber attachment plate; would he still use sulphuric acid, or would he use the potassium solution? He suggests the cleaning of metal plates with sulphuric acid.

*Dr. James Truman.*—The paper is interesting, especially in that part of it where Dr. Nones says we must submit ourselves to circumstances. The iron-clad rule which has so long prevailed with dentists of extracting all teeth is, I think, wrong. If two or three teeth are in the mouth, they will remove these without taking into consideration the health of the patient or the conditions that may supervene. In my opinion the extraction of teeth and even the extraction of roots for the purpose of insertion of artificial dentures has been carried too far. We do not take into consideration sufficiently that these roots are of great value in mastication. Take, for instance, a case where we have two laterals in the mouth with the central roots between. Now, most dentists would take them out. I would advise their proper treatment and retention as the roots furnish a base for mastication which the gum cannot.

During the past winter I instructed a student to make a denture over just such a case as I have cited. He was astonished, and could hardly persuade himself to make the set, and asked the opinion of several others. Their judgment was that the roots should all come out. What were the conditions? Here was an elderly woman, very feeble and unable to bear the extraction of the roots. Taking these facts into consideration, I ordered the plate made, which was done after treating the roots.

The idea of extracting teeth is too common. We must treat our patients as we find them; not as we would like to have them. It is much easier to make a denture where all teeth are extracted; but there are many cases where they are very valuable for clasping; and in the lower jaw, especially, it becomes of vital importance that they be preserved to give stability to the artificial teeth.

I am glad that this question has been brought up. In former years it was taught that the mouth must be freed entirely of roots. I do not believe that now. I do hold that the European idea of keeping all the roots in is a very bad one, especially considering the state in which they generally keep them. We must make everything healthy before the impression is taken, or subsequently. It is very difficult to keep the roots in a healthy condition after the plate is inserted, but by the use of proper antiseptics they may be kept in a condition sufficiently satisfactory to warrant retention.

With regard to the production of irritation and inflammation by plates, several years ago Dr. Black demonstrated that vulcanite plates are very fine culture-fields for micro-organisms, and that these really produce the irritation. You know that in former years dentists supposed that it was attributable to the vermilion in the vulcanite. A committee was appointed many years ago to examine into this, and demonstrated quite conclusively that there was no free mercury upon the surface and that the irritation could not be due to that. The idea that it was the non-conducting property of the rubber that produced it was then very general. I question that very much, although, perhaps, it has something to do with it, but it can only be tested by the determination of the increase of temperature between the plate and the mucous membrane. It is probably altogether owing to the development of micro-organisms upon the surface of the vulcanite plates, because these are almost invariably rough. If they were polished upon their contact surfaces I doubt very much if there would be any difficulty. If the patients were directed to use a powerful antiseptic there would probably be no trouble from inflamed gums.

The plate should be taken out and placed in an antiseptic wash over night, and there is nothing better for this than hydronaphtol solution. Why porcelain plates are not affected in the same way as rubber plates I cannot understand, because they are not very well polished; and the roughness here should make a good place for the development of micro-organisms.

*Dr. Hickman.*—When I first began to practise, I thought it was against all rules to make a denture over roots. In fact, I thought I had been taught that. One of my first patients wanted me to make a plate over at least eight or nine roots. She had been wearing a denture, and I told her I would not do it. I lost my patient by refusing to do so. In about two months she died of tuberculosis. I have thought that by giving my experience my conscience would be easier. I feel that if I had done as Dr. Nones advises I should feel much better about it.

I would like to know if the use of thin tin-foil over the model, as we are now doing at the University, would not entirely do away with the pin-point roughness.

*Dr. I. N. Broomell.*—I have always looked upon vulcanite as irritating, partly because it so accurately fits the parts. If we compare the surface of a vulcanite plate with that of a gold or other metal plate, particularly under a microscope, we will find that the latter is absolutely smooth, while the former is rough. As Dr. Nones has said, these invisible pin-points are, no doubt, the source of irritation. Not only these pin-points to which he has referred, but also the sharp lines produce irritation. These, of course, can be polished, and I instruct my students to polish the plate sufficiently to remove all fine prominences.

I would ask Dr. Nones one question. He referred to the surgical treatment of double lip. I would like to know the surgical treatment.

*Dr. H. E. Roberts.*—While fitting a vulcanite plate to the mouth the patient almost invariably asks, "Shall I wear this all the time and wear it at night?" My own advice to the patient is, "Leave your plate out at night if you can possibly do so. If it makes your jaws ache so that you cannot get along without it, then wear it; otherwise leave it out as much as possible." The mucous membrane beneath a vulcanite plate becomes inflamed. The plate fitting as accurately as it does acts like a bandage on any other part of the body. The skin becomes tender and soft under the bandage, and the mucous membrane under the plate gets into the same condition.

Dr. Nones made no reference in his paper to the subsequent care of the mouth,—that is, to keeping it clean and in a healthy condition. I think that the removal of the plate at night is a very important means to this end.

*Dr. Huey.*—Will Dr. Roberts explain whether he finds as much irritation from the metal plates as he does from the vulcanite.

*Dr. Roberts.*—I never find as much irritation under a metal plate as I do under a vulcanite one. Under the latter the mucous membrane becomes hot, because it fits so accurately, while with a metal plate relief is obtained through the air-spaces. Every surface should have a rest. I think the keeping in of the plate at night is about the same as wearing shoes in bed.

*Dr. Coney.*—There is one plate that will not cause the mucous membrane to become inflamed. That is the porcelain, and it is the only plate that is absolutely cleanly in the mouth. I do not think a vulcanite plate can be made to fit tighter, but I have seen gums very much inflamed by vulcanite, but not by porcelain. I have taken out vulcanite plates and also metal ones and found inflammation present. I have put in porcelain plates and afterwards the mouths became hard and healthy. I had a case of that kind in Princeton some seventeen years ago, where vulcanite plates had been worn for a number of years. After the patient had worn one for a year the tissues became so soft that he could not keep his plate up and had to remove it every night. I made a porcelain plate for him, which he wore for about six weeks. I reground it, and he wore it for six months. I then made another one which he wore a year. Then I made him another, and that was the last, and I believe he wore it up to the time of his death, which occurred some years afterwards. I have seen porcelain plates that have been worn for twenty-five or thirty years, but I have never seen irritation produced by them. Why should it be necessary to take the plate out at night? Would it not be better for the patient to keep the plate in the mouth for rest?

*Dr. M. J. Schamberg.*—We have heard numerous explanations of the cause of inflammation from artificial dentures, and in trying to sum up I have come to the conclusion that each statement is very plausible, and that, therefore, we ought to classify. I think we can with justice say that the cause of the inflammation of the mucous membrane is not alone the non-conductivity of the plate. We ought to take into consideration all of the conditions that may exist. In the first place, we know that a loosely-fitted plate is very likely to produce irritation; that we have ulcers due to putrefactive changes which are accountable to the food which collects on the plate; then we may have a plate which fits entirely too tightly. If you place in an artificial denture which causes continual and excessive pressure, you are bound to have inflammation of the underlying parts. A patient should remove a plate at night for the same reason that he should take off his rubber shoes upon coming within doors. If rubber shoes are worn continuously they cause the feet to become tender and inflamed. A vulcanite plate acts in practically the same manner.

*Dr. H. C. Register.*—Dr. Nones is an expert, and has given us this evening a practical paper. I think, however, that the discussion is digressing from the subject of his paper, which, if I understand correctly, is the preparation of the mouth for artificial dentures. If any man knows how to do that, I think Dr. Nones does, yet there were one or two points which he seems to have omitted which have been of great moment to me. The impression upon which we are going to form the future denture is an essential to success, and the subcutaneous tissue must be considered in order to obtain usefulness of a denture. I think that most operators believe that plaster of Paris always gives the best results. We get a fac-simile of the mouth upon which to form the denture, and from this make a negative which must be a success. In some cases the tissue is of such a character that it must act differently with the plate in the mouth from what it does with the plate out of the mouth. In other words, we have a soft condition of the mucous membrane, physically healthy and yet springy. I have found in these cases that it was absolutely impossible to get a perfectly satisfactory denture made from an impression taken in plaster of Paris, while with the modelling compound, particularly the hard variety, these cases would be invariably successful. I merely call attention to that. It is a little point, yet a great one for comfort to the patient.

Another point to which Dr. Nones called our attention is the retention of as many teeth as can be made useful either for mastication or for retention of the plate. Since I have grown old I have retained one bicuspid upon which I do a great deal of work, and I never appreciated until recently how much work could be done by one or two natural teeth in the mouth. I think that every operator in preparing the mouth should be extremely careful to preserve all teeth that can be made useful. A great many of these teeth and roots, and roots that cannot be crowned, can be made healthy with a very little treatment. As a mouth-wash, one of the best things that I know of is sea-salt in solution. Dissolve a pinch of it in a little water and wash out the throat and nares. There is nothing more stimulating to mucous membrane or destructive of germ-life. There are many persons wearing artificial teeth who do not appreciate how filthy the mouth can become.

Many years ago I inserted an obturator covered with a gold plate. The patient went away, and I do not think that I saw him again for six or eight years, when he informed me that the plate had never been removed during that time. He said he was afraid he would never be able to get it in again.

*Dr. M. H. Cryer.*—The condition referred to as double lip is not exactly double lip, but a fold of mucous membrane lapping over a badly fitting plate, or one that has been kept in. The irritation starts upon the upper edge of the membrane and folds over and over. I have seen the growth in some cases protruding below the true lip. I had a marked case while Dr. Nones was a student at the Philadelphia Dental College. It was dissected away with a scalpel, and a few stitches, perhaps four on each side, put in to bring the mucous membrane of the lip into apposition with the membrane covering the alveolar process. The operation was successful and the patient recovered quickly. In some cases, where not very marked, it can be treated with an alum solution or some other astringent.

With regard to the removal of teeth and roots. I was taught to remove all roots that a plate would cover. I believe it is the general practice and a good one, but there may be exceptions. The great objection to allowing a root to remain is that the plate rests upon it, the gum grows over the edge, and becomes irritated and hypertrophied. In my teaching I recommend, as a rule, the extraction of roots which will lie beneath the plate; but in exceptional cases, if a root can be kept in a perfectly healthy condition, I would let it remain.

I believe that it is better to remove a plate at night, for that gives the tissues a perfect rest, and the parts are then ready the next day to sustain the plate in position. If a plate is constantly kept in the mouth the vacuum-chamber is filled with the mucous membrane and the plate does really no good. The large majority of mechanical dentists use a larger vacuum-chamber in each succeeding plate until, as a result, the mouth may not only be level from side to side, but the centre of the mouth on a level with the alveolar process. I think that by keeping the plate out of the mouth at night the parts will be induced to return to their normal condition, and the deformity in the centre of the mouth will not be produced.

*Dr. Nones.*—In answer to Dr. Hickman's question, I would say that I simply clean the rubber attachment plate with pumice and not with acid.

With reference to the use of tin-foil to avoid roughness of the vulcanite, you will remember that I said it could be done by the use of tin dies, but even then some spots will be found on a vulcanite plate.

As to the double lip, Dr. Cryer answered that question. It is not a scientific name, but it is an easy one for me. I have seen many cases of that kind, but very few as marked as the one of which Dr. Cryer spoke. I saw the operation upon that one. The beginning of this condition may frequently be observed in cases of tightly fitting vulcanite plates.

I fully agree with Dr. Roberts in his position, and always tell my patients to remove their dentures at night, believing that if the advice is not satisfactory to them they will do as they please, while for my part I feel that I can do no harm by telling them to remove the plate. In most cases more harm than good is done by leaving it in.

I hardly think that irritation is caused by a tight fit, but, as Dr. Truman remarked, the vulcanite plate seems to be a favorable locality for micro-organisms, the germs thrive beneath it. I have seen both tightly fitting and very loosely fitting plates produce this condition.

Replying to Dr. Coney's question, I may say that I have seen porcelain plates also produce irritation, but not in many cases, for one does not see many porcelain plates. I think it is not nearly so likely to happen under porcelain as under vulcanite, and it may be explained that germs do not thrive upon a porcelain plate. Nature seems to be friendly to porcelain. You may make a porcelain crown and poorly fit it to the root, and the gum will adapt itself to it and really not be irritating, while again you may fit a metal crown in the same way and have the same gum irritated.

My paper being upon the preparation of the mouth alone, I made no reference to the preparation of the plate nor to the taking of impressions in soft, flabby conditions.

Adjourned.

Otto E. Inglis,
*Editor Academy of Stomotology.*

